# Autologous Plasma Gel as a Dermal Filler: A Head-to-Head Experimental Comparison with Hyaluronic Acid Fillers and Fat Grafts in a Rat Model

**DOI:** 10.1007/s00266-026-06041-5

**Published:** 2026-06-30

**Authors:** Oguz Atan, Murat Celik, Nezih Sungur, Kadri Ozer, Hakan Teymur, Gokay Baykara, Muzaffer Caydere, Sema Hucumenoglu, Pinar Nercis Kosar, Ugur Kocer

**Affiliations:** 1 Department of Plastic, Reconstructive and Aesthetic Surgery, Ankara Training and Research Hospital, Ankara, Turkey; 2 Private Practice, Ankara, Turkey; 3 Department of Plastic, Reconstructive and Aesthetic Surgery, Sivas Cumhuriyet University, 58070 Sivas, Turkey; 4 Department of Plastic, Reconstructive and Aesthetic Surgery, Istanbul Florence Nightingale Hospital, Istanbul, Turkey; 5 Private Practice, Istanbul, Turkey; 6 Department of Pathology, Ankara Training and Research Hospital, Ankara, Turkey; 7 Department of Radiology, Ankara Training and Research Hospital, Ankara, Turkey

**Keywords:** Plasma gel, Autologous filler, Dermal filler, Hyaluronic acid, Fat grafting, Biocompatibility, Animal model

## Abstract

**Background:**

Dermal fillers are widely used in aesthetic practice; however, none fully meet expectations in terms of biocompatibility, predictability, and long-term stability. Autologous plasma gel (PG) has been proposed as a biological alternative, yet comparative experimental data remain limited.

**Objective:**

To evaluate PG as a dermal filler and compare its volumetric behavior and histopathological characteristics with hyaluronic acid fillers and autologous fat graft (FG) in a rat model.

**Methods:**

Thirty-two male Sprague–Dawley rats were included. In a within-subject design, PG, FG, and two commercial hyaluronic acid fillers (HA-1 and HA-2) were injected into predefined dorsal quadrants. Volumetric assessment was performed using computed tomography at 1, 3, and 5 months. Histopathological evaluation focused on material persistence, neovascularization, and inflammatory response.

**Results:**

At 1–3 months, PG demonstrated lower volume compared with the other materials. By 5 months, no significant differences were observed. Despite lower early volume, PG exhibited a more stable volumetric profile over time compared with FG and became comparable to hyaluronic acid fillers at later time points. Histologically, PG showed lower inflammatory and neovascularization scores, while material persistence remained similar across groups.

**Conclusions:**

PG demonstrated favorable biocompatibility and a stable longitudinal volume profile in this experimental model. Although early volume was lower, long-term outcomes were comparable to established fillers, supporting further clinical investigation of PG as an autologous filler option.

**Level of Evidence V:**

This journal requires that authors assign a level of evidence to each article. For a full description of these Evidence-Based Medicine ratings, please refer to the Table of Contents or the online Instructions to Authors www.springer.com/00266.

**Supplementary Information:**

The online version contains supplementary material available at 10.1007/s00266-026-06041-5.

## Introduction

Minimally invasive facial rejuvenation procedures have become an integral part of contemporary aesthetic practice, driven by patient demand for effective volume restoration with minimal recovery time and low complication rates [1, 2]. Among injectable materials, hyaluronic acid–based fillers and autologous fat grafting remain the most widely utilized modalities [3, 4]. Despite their established clinical roles, both approaches present inherent limitations related to cost and predictability.

Autologous fat grafting offers volumetric augmentation together with regenerative potential; however, its clinical reliability is frequently unpredictable due to resorption rates and donor-site morbidity [5, 6]. These limitations may reduce procedural consistency, particularly in aesthetic indications where precise volume control and reproducibility are critical. Hyaluronic acid fillers, while providing more predictable short-term volumetric outcomes, are associated with higher costs, reliance on exogenous materials, and the potential for inflammatory or foreign body reactions, especially in anatomically sensitive regions [7, 8].

In daily practice, there remains an unmet need for an autologous, cost-effective, and biologically compatible filler material capable of providing reproducible volumetric outcomes with minimal tissue reaction. In this context, autologous plasma gel (PG) has recently emerged as a potential biological filler [9, 10]. Derived from platelet-poor plasma via controlled thermal processing, PG is an acellular, injectable hydrogel of autologous origin with favorable handling characteristics. Preliminary clinical and experimental reports have suggested its feasibility as a temporary volumizing agent; however, objective experimental data directly comparing PG with established filler materials under standardized conditions remain limited [6, 10]. Importantly, existing studies evaluating PG have predominantly focused on isolated comparisons or short-term outcomes, often lacking direct head-to-head analysis against multiple commercially available hyaluronic acid fillers and autologous fat grafting within the same experimental model. Consequently, the relative volumetric behavior, tissue response, and inflammatory profile of PG compared with widely accepted filler standards remain incompletely characterized.

The present experimental study was designed to address this gap by performing a controlled, head-to-head comparison of PG, FG, and two commonly used commercial hyaluronic acid fillers within the same animal model. By evaluating longitudinal volumetric changes using radiologic measurements together with blinded histopathological assessment of tissue response, this study aimed to determine whether PG demonstrates volumetric stability and biocompatibility comparable to established filler materials over extended follow-up periods.

## Materials and Methods

### Study Design and Ethical Approval

This controlled experimental animal study was approved by the local Animal Ethics Committee (Approval No: 0041/481) and conducted in accordance with institutional and international guidelines for the care and use of laboratory animals.

### Animal Model

A total of 32 male Sprague–Dawley rats (aged 2–4 months, weighing 200–250 g) were included in the study. Five animals were used as donors for the preparation of PG and autologous fat grafts. The remaining 27 animals were randomly assigned to three experimental groups (*n* = 9 per group) according to predetermined sacrifice time points at 1, 3, and 5 months. All animals were maintained under standardized laboratory conditions throughout the study.

### Filler Materials

Four different filler materials were evaluated: PG, FG, and two commercially available hyaluronic acid fillers (HA-1 and HA-2). PG and FG were prepared from donor animals under standardized conditions. HA-1 (Juvederm®, Allergan Aesthetics, Irvine, CA, USA) and HA-2 (Teosyal®, Teoxane Laboratories, Geneva, Switzerland) were used as representative hyaluronic acid–based fillers commonly applied in clinical practice. These materials were selected to enable a direct comparison between autologous biological fillers and commercially available hyaluronic acid products with differing rheological characteristics.

### Preparation of PG

**Fig. 1:**
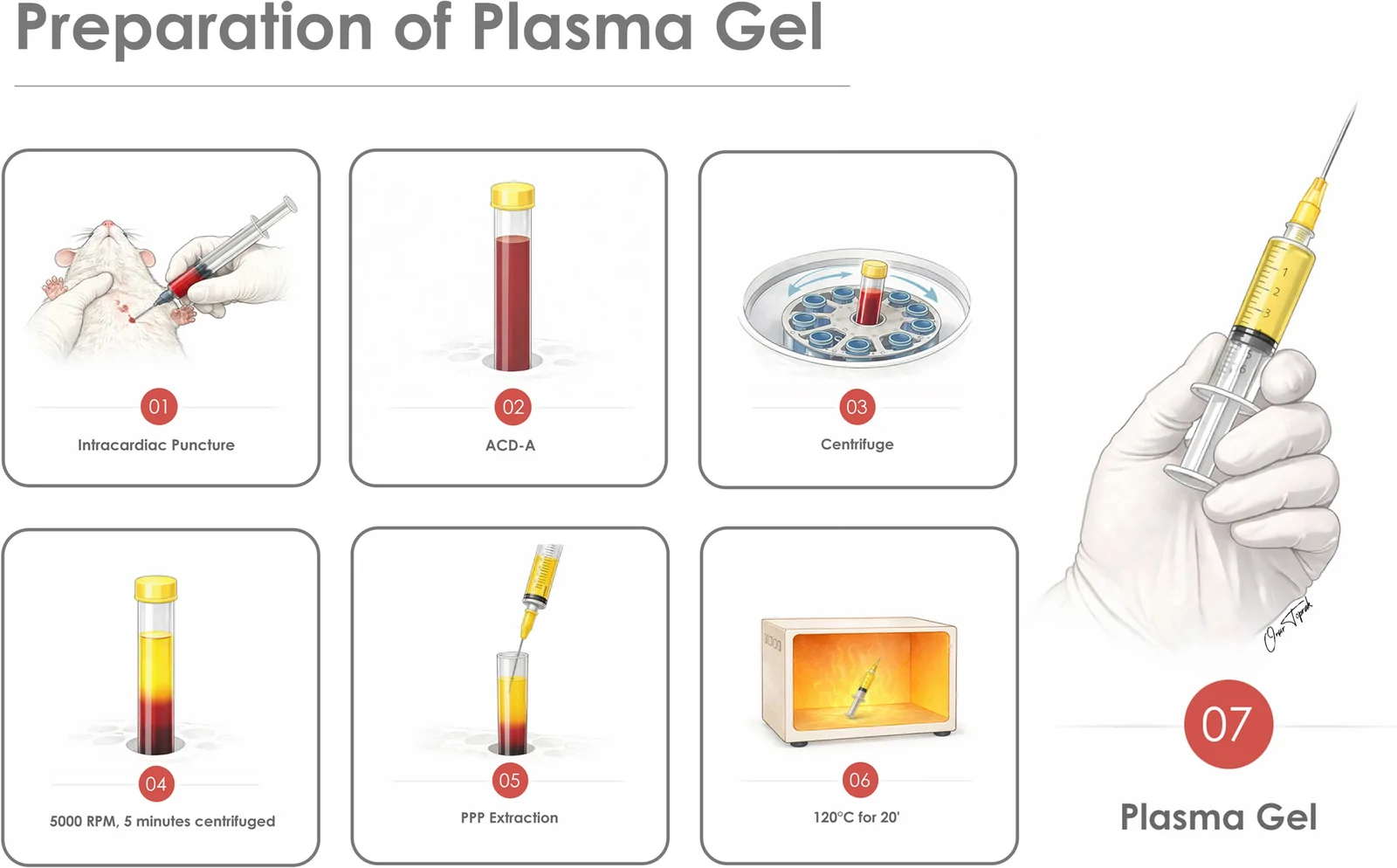
Experimental design and preparation of autologous plasma gel (PG). Stepwise preparation of PG. Whole blood was collected and mixed with anticoagulant acid-citrate-dextrose solution (ACD-A), followed by centrifugation. Platelet-poor plasma (PPP) was separated and thermally processed at 120 °C for 20 minutes to obtain PG prior to injection

PG was prepared under sterile conditions using whole blood obtained via intracardiac puncture from donor animals under general anesthesia. Approximately 8–10 mL of blood per rat was collected into tubes containing acid-citrate-dextrose solution (ACD-A) at a ratio of 1:9. Samples were centrifuged at 5000 rpm for 15 minutes to obtain platelet-poor plasma. The plasma fraction was carefully aspirated and subjected to controlled thermal processing at 120 °C for 20 minutes using an autoclave, resulting in a homogeneous, acellular, and injectable hydrogel (Fig. 1).

No exogenous activators, cross-linking agents, calcium salts, or thrombin were used. The final product was used immediately after preparation.

### Preparation of FG

FG was harvested from the inguinal fat pads of donor rats. Approximately 1.5 mL of adipose tissue per donor was obtained and mechanically processed using a slicing technique prior to injection.

### Injection Protocol

**Fig. 2:**
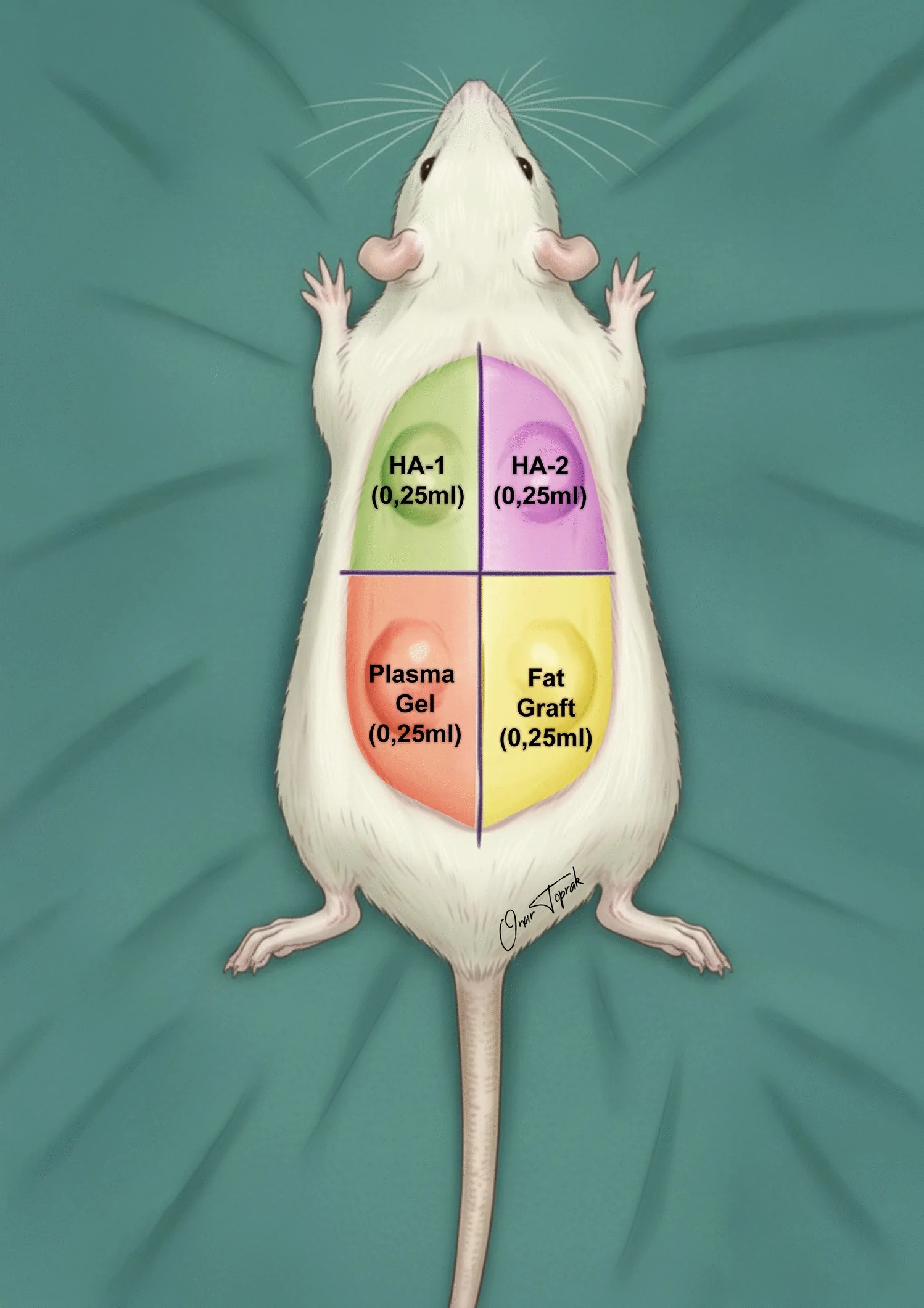
Injection protocol. Schematic illustration of the dorsal injection model in rats. Each animal received four subcutaneous injections (0.25 mL each) into separate dorsal quadrants: autologous plasma gel (PG), autologous fat graft, and two commercially available hyaluronic acid fillers (HA-1 and HA-2)

Following randomization, the dorsal region of each animal was divided into four equal quadrants. Each rat received four subcutaneous injections (0.25 mL per site) as follows: upper left quadrant, HA-1; upper right quadrant, HA-2; lower left quadrant, PG; and lower right quadrant, FG (Fig. 2). Injection sites were standardized across all animals. This within-subject design enabled direct head-to-head comparison of all materials under identical biological conditions while minimizing inter-animal variability.

### Volumetric Assessment

**Fig. 3:**
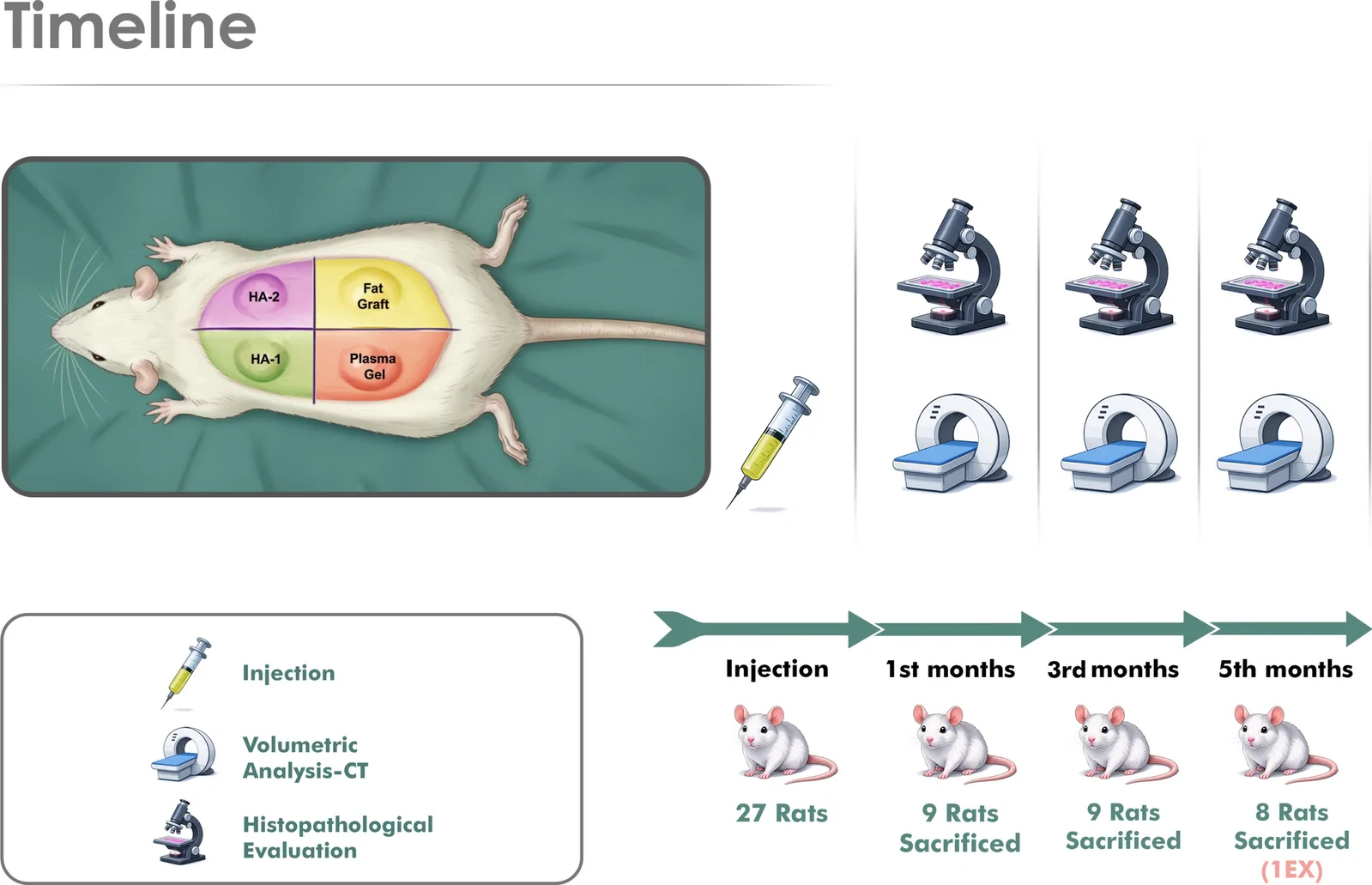
Experimental timeline and evaluation methods. Schematic overview of the experimental timeline showing injection and follow-up evaluations at 1, 3, and 5 months. Volumetric analysis was performed using computed tomography, followed by histopathological evaluation at each sacrifice time point

Volumetric evaluation was performed using computed tomography (CT) as the primary outcome measure at 1, 3, and 5 months prior to sacrifice (Fig. 3). Early postoperative measurements were intentionally excluded to avoid confounding effects related to injection-induced edema and immediate post-injection volume changes. CT imaging was performed using a high-resolution system (55 kV tube voltage, 450 μA tube current). Volumetric measurements were obtained using voxel-based analysis within predefined regions of interest.

### Histopathological Evaluation

At the predetermined sacrifice time points (1, 3, and 5 months), following imaging and documentation, the dorsal tissues were excised en bloc and fixed in 10% neutral-buffered formalin. After paraffin embedding, representative sections (4–5 μm) were obtained from each injection site and stained with hematoxylin–eosin.

Histopathological evaluation was performed by a blinded pathologist. The following parameters were assessed using a semi-quantitative ordinal scale ranging from 0 to 3 (0 = absent, 1 = mild, 2 = moderate, 3 = marked): material persistence; neovascularization (based on the number and distribution of newly formed vascular structures within and around the injected material); and inflammatory response (evaluated by the presence and density of inflammatory cell infiltrates, including lymphocytes, macrophages, and foreign body giant cells).

### Statistical Analysis

Statistical analyses were performed using SPSS software (version 20.0). Data distribution was assessed using the Kolmogorov–Smirnov test. For normally distributed variables, intergroup comparisons were performed using one-way ANOVA, while nonnormally distributed variables were analyzed using the Kruskal–Wallis test. A *p* value < 0.05 was considered statistically significant.

## Results

A total of four groups were analyzed: PG, FG, HA-1, and HA-2. All injections were performed using a standardized volume of 0.25 mL per site. Volumetric assessment was performed using computed tomography (CT) at 1, 3, and 5 months postoperatively. Detailed volumetric data are presented in Table 1.

**Table 1:** CT-based volumetric evaluation of injected materials at different time points

Time point	FG	PG	HA-1	HA-2	*p*-value
1 Month	151.97 ± 26.99 (131.22–172.72)	104.49 ± 5.61 (100.17–108.80)	141.02 ± 26.28 (120.82–161.22)	127.56 ± 14.91 (116.10–139.02)	< 0.001
3 Months	100.88 ± 23.22 (83.03–118.73)	78.95 ± 16.86 (65.99–91.91)	112.40 ± 21.73 (95.69–129.10)	119.19 ± 28.06 (97.62–140.76)	< 0.01
5 Months	35.39 ± 13.35 (10.34–51.69)	26.07 ± 5.56 (16.17–31.92)	36.30 ± 11.70 (11.01–50.01)	42.91 ± 16.06 (13.44–66.12)	> 0.05

^*^Values are expressed as mean ± standard deviation (minimum–maximum). Statistical comparisons were performed using appropriate non-parametric or parametric tests as indicated. A *p*-value < 0.05 was considered statistically significant. *FG* fat graft; *PG* plasma gel; *HA-1* hyaluronic acid filler 1 (Juvederm®); *HA-2* hyaluronic acid filler 2 (Teosyal®)

### Volumetric Outcomes

**Fig. 4:**
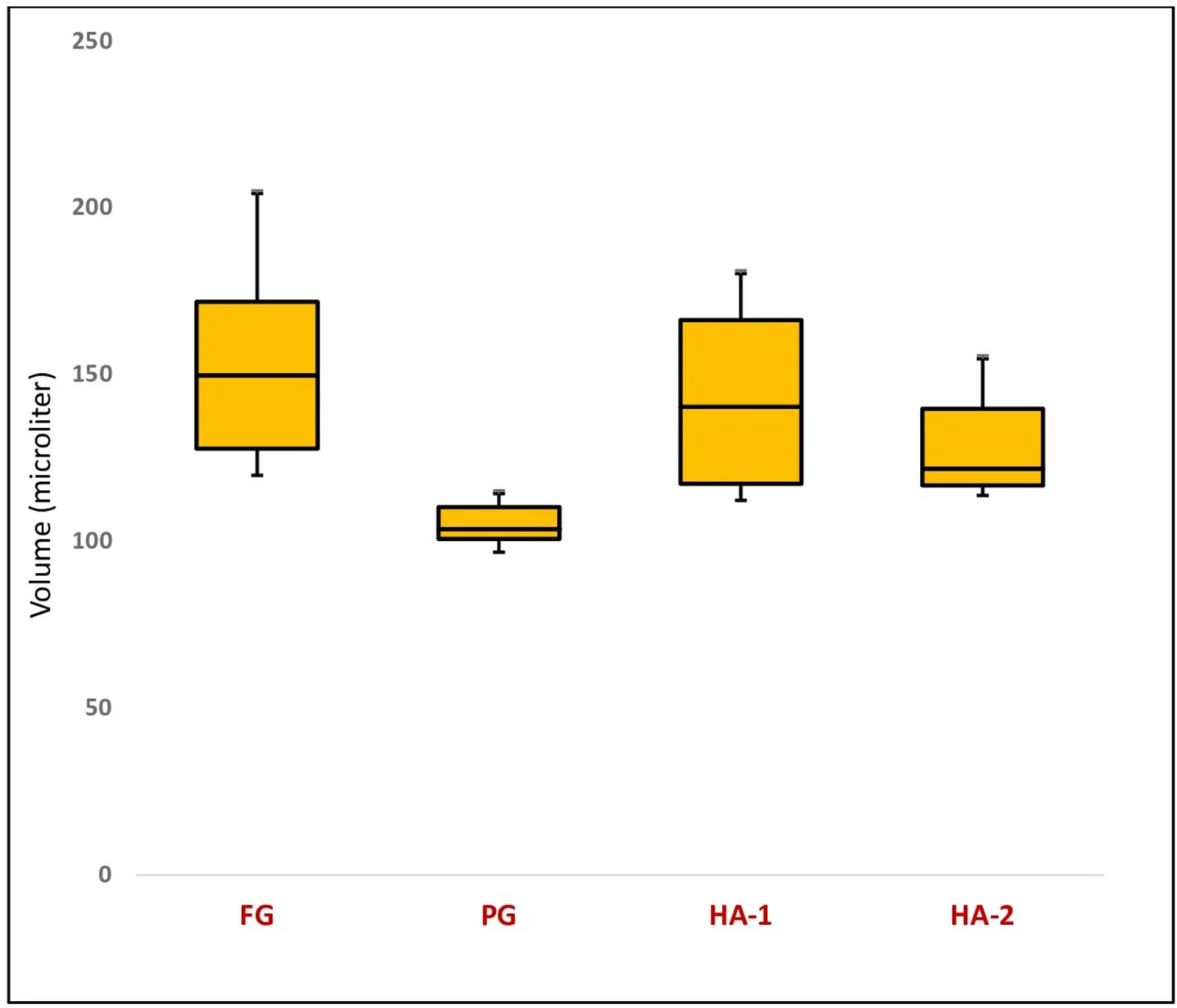
Interquartile comparison of volumetric measurements across study groups at 1 month. Data are presented as minimum, 25th percentile, median, 75th percentile, and maximum values. A statistically significant difference was observed among the groups (Kruskal–Wallis test, X2 = 21,430, *p* < 0.001). FG: fat graft group; PG: plasma gel group; HA-1: hyaluronic acid group 1; HA-2: hyaluronic acid group 2

At 1 month, significant differences in volume measurements were observed among the groups (*p* < 0.001) (Fig. 4). The FG group demonstrated the highest mean volume, followed by HA-1 and HA-2, whereas PG showed the lowest volume values. Pairwise comparisons revealed a statistically significant difference between FG and PG (*p* < 0.05). In contrast, no significant differences were observed between PG and either HA-1 or HA-2, nor between HA-1 and HA-2 (*p* > 0.05).

**Fig. 5:**
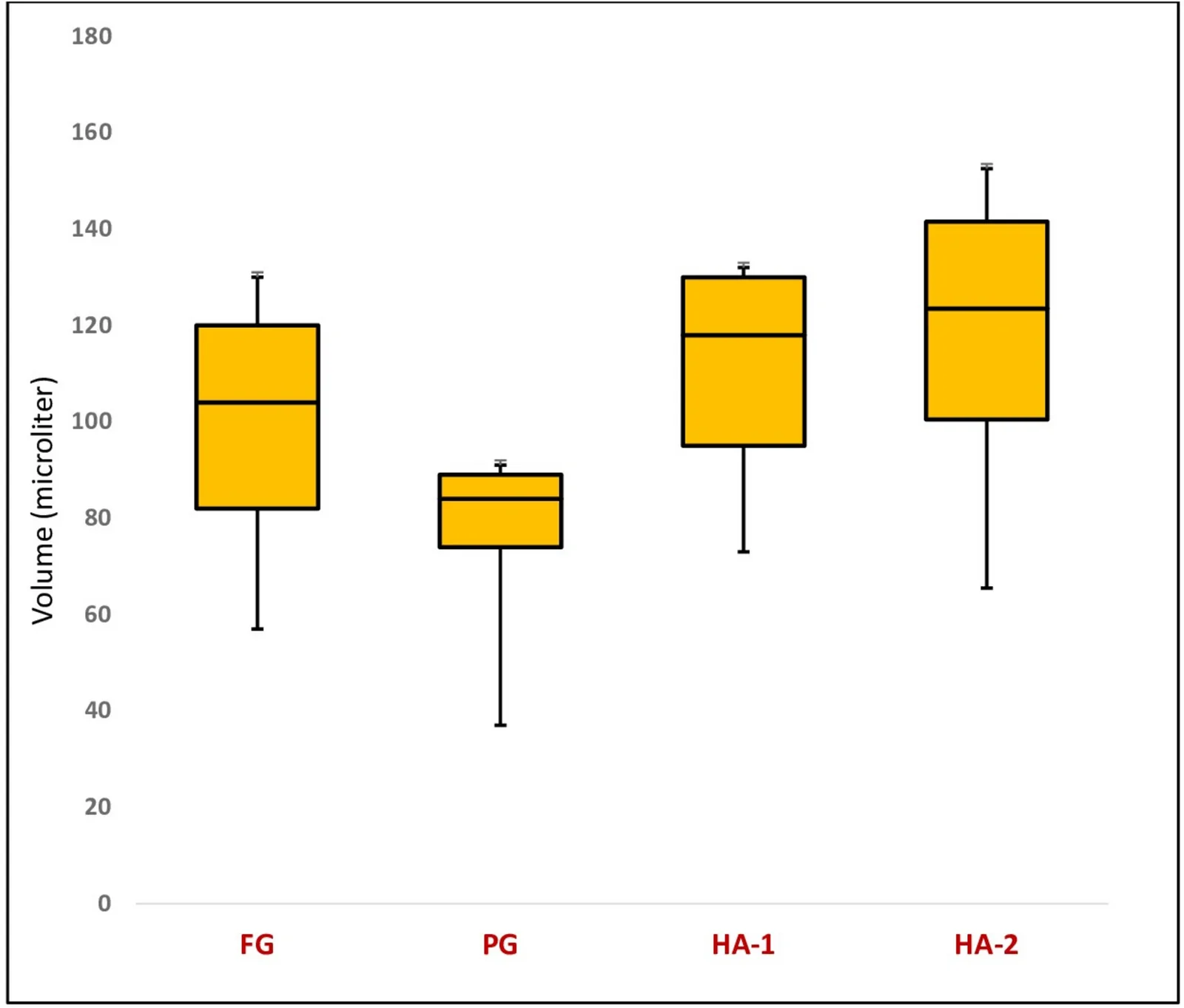
Interquartile comparison of volumetric measurements across study groups at 3 months. Data are presented as minimum, 25th percentile, median, 75th percentile, and maximum values. A statistically significant difference was observed among the groups (Kruskal–Wallis test, X2 = 10,179, *p* < 0.05). FG: fat graft group; PG: plasma gel group; HA-1: hyaluronic acid group 1; HA-2: hyaluronic acid group 2

At 3 months, intergroup differences remained statistically significant (*p* < 0.01) (Fig. 5). PG demonstrated significantly lower volume compared with all other groups (*p* < 0.05 for all comparisons). No significant difference was observed between FG and either HA-1 or HA-2 (*p* > 0.05), and HA-1 and HA-2 remained comparable to each other.

**Fig. 6:**
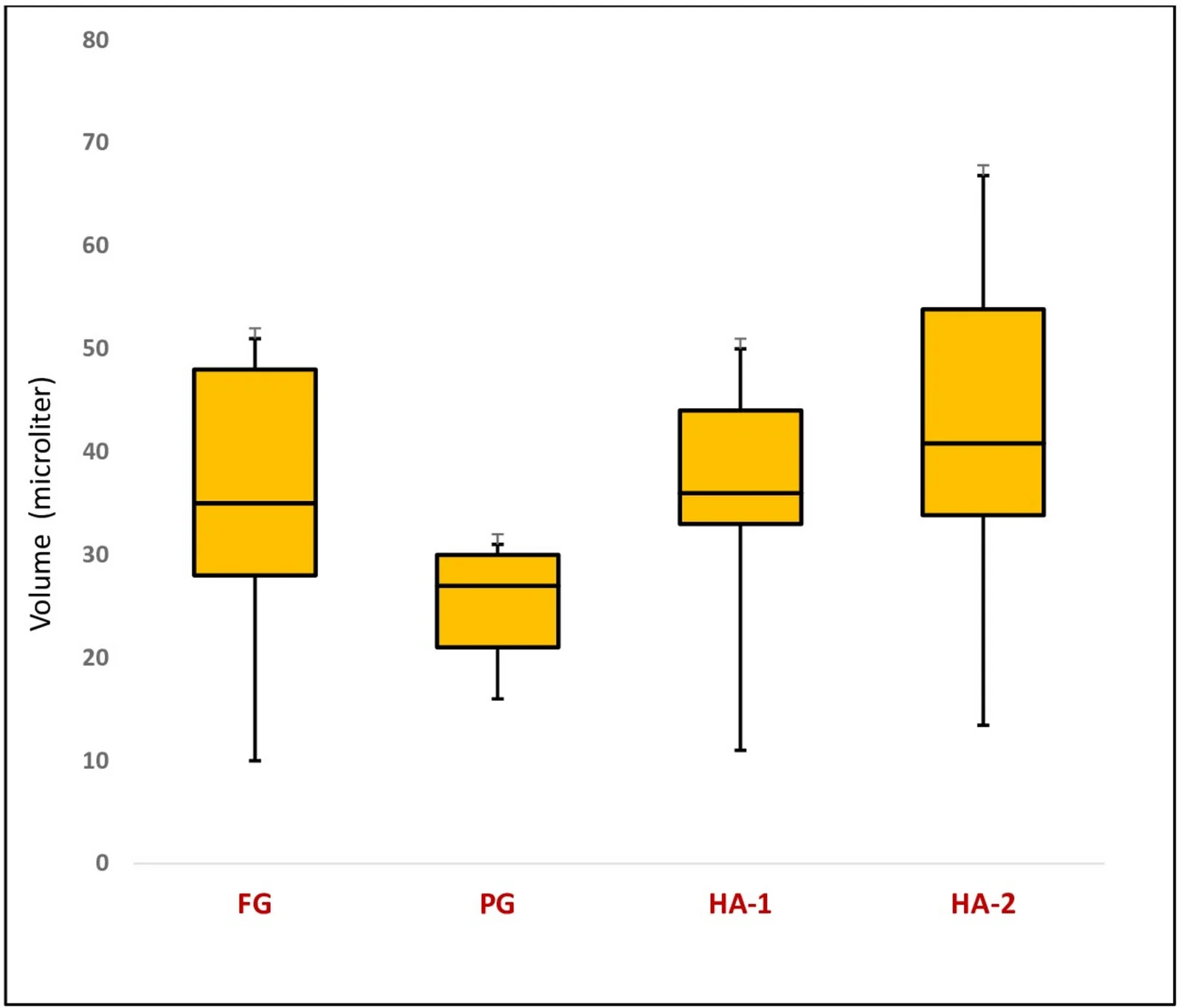
Interquartile comparison of volumetric measurements across study groups at 5 months. Data are presented as minimum, 25th percentile, median, 75th percentile, and maximum values. A statistically significant difference was not observed among the groups (Kruskal–Wallis test, X2 = 9058, *p* > 0.05). FG: fat graft group; PG: plasma gel group; HA-1: hyaluronic acid group 1; HA-2: hyaluronic acid group 2

At 5 months, no statistically significant differences in volumetric measurements were observed among the groups (*p* > 0.05) (Fig. 6).

**Fig. 7:**
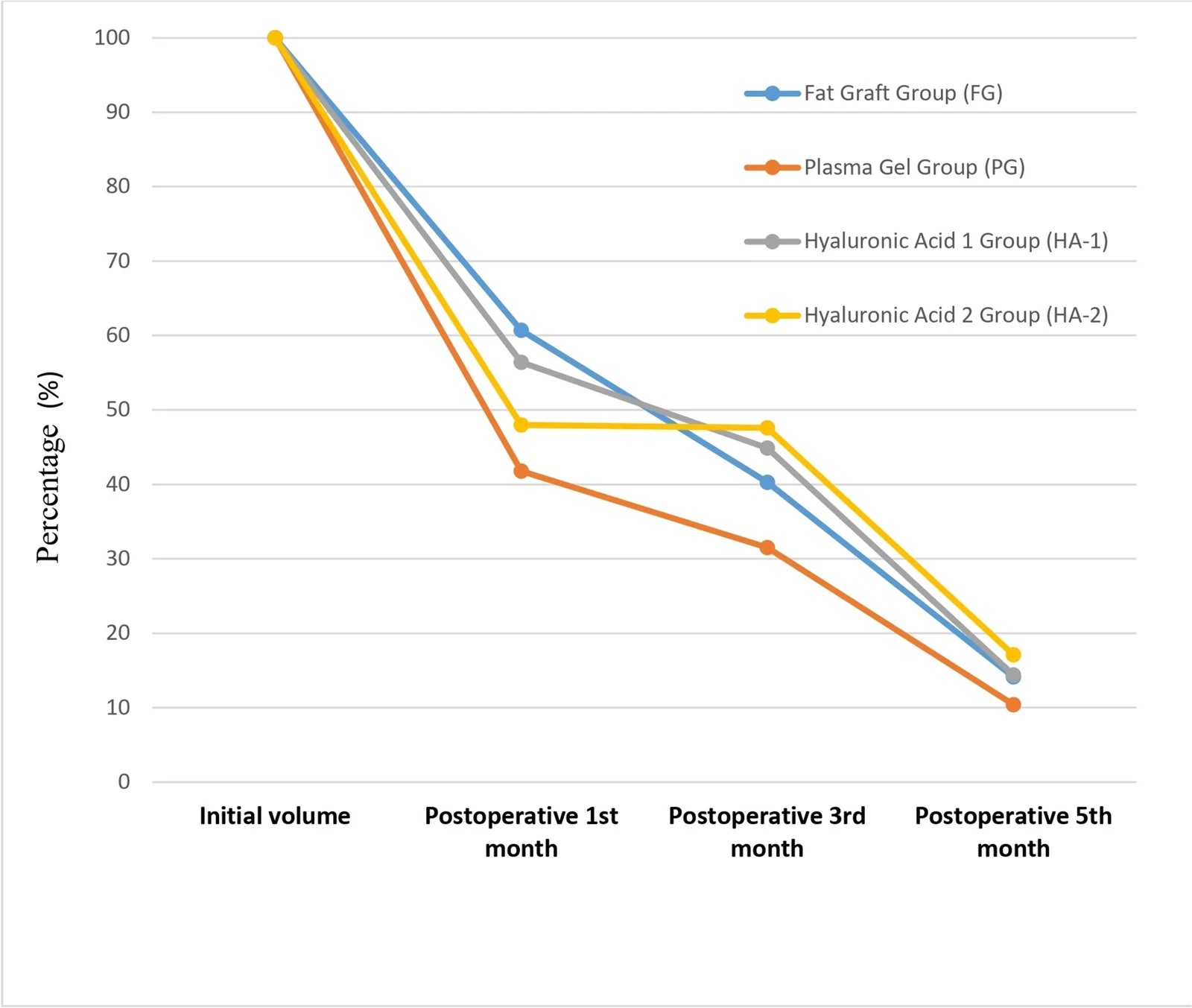
Percentage of volume retention relative to initial injected volume across study groups and time points

Longitudinal analysis of volume retention demonstrated a progressive decline across all groups over time, with comparable residual volumes observed at 5 months (Fig. 7; Table 1).

### Histopathological Findings of “Material Persistence”

**Fig. 8:**
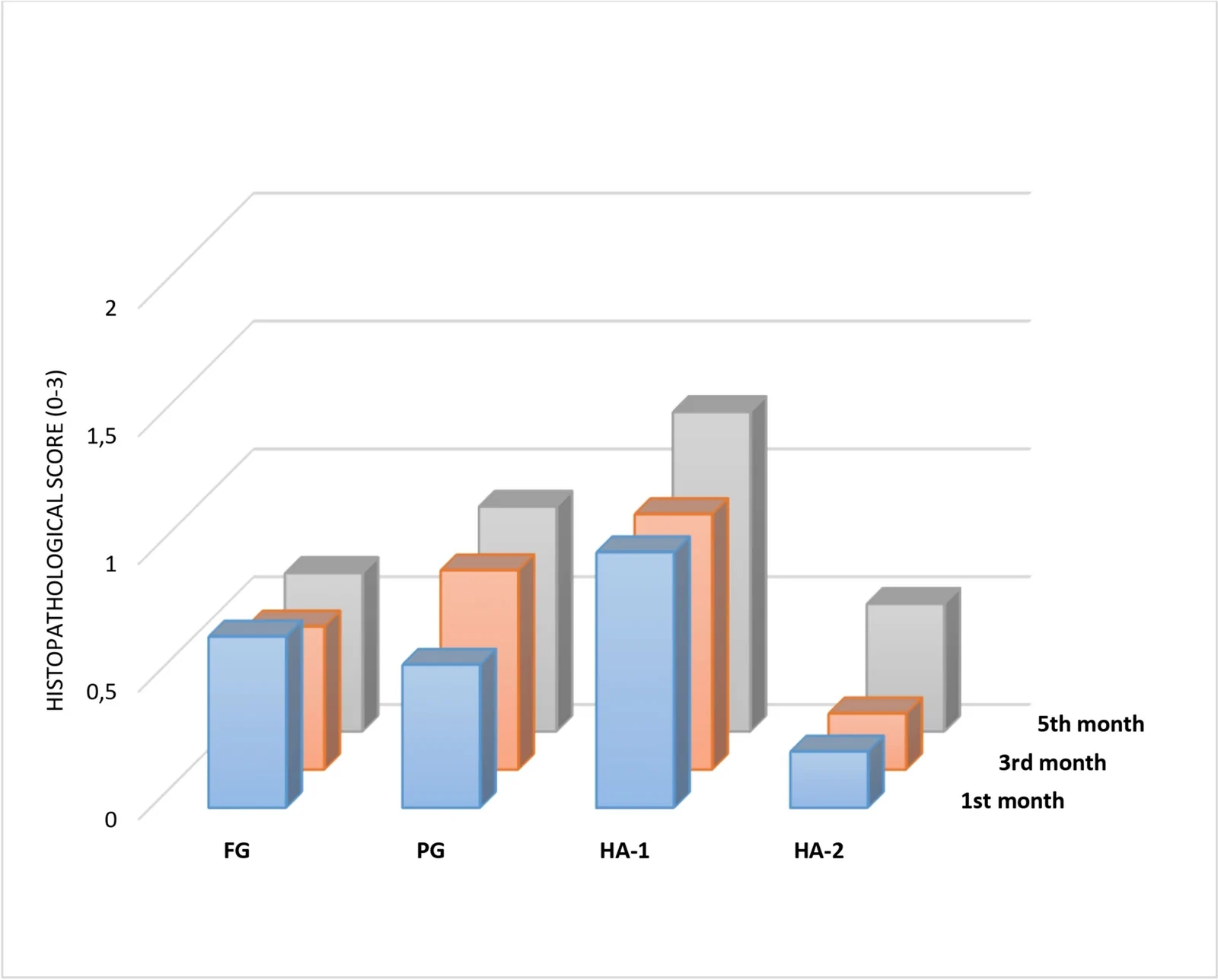
Comparison of histopathological “material persistence scores” across study groups at different time points

At all-time points (1, 3, and 5 months), no statistically significant differences were observed among PG, FG, HA-1, and HA-2 in terms of material persistence (*p* > 0.05 for all comparisons) (Fig. 8). Detailed data are presented in Table 2.

**Table 2:** Histopathological evaluation of material persistence at different time points

Time point	FG	PG	HA-1	HA-2	*p*-value
1 Month	0.67 ± 0.86 (0–2)	0.56 ± 0.52 (0–1)	1.00 ± 1.22 (0–3)	0.22 ± 0.66 (0–2)	> 0.05
3 Months	0.56 ± 0.52 (0–1)	0.78 ± 0.67 (0–2)	1.00 ± 1.32 (0–3)	0.22 ± 0.44 (0–1)	> 0.05
5 Months	0.62 ± 0.52 (0–1)	0.88 ± 0.64 (0–2)	1.25 ± 1.16 (0–3)	0.50 ± 0.53 (0–1)	> 0.05

^*^Values are expressed as mean ± standard deviation (minimum–maximum). Statistical comparisons were performed using appropriate non-parametric tests as indicated. A *p*-value < 0.05 was considered statistically significant. *FG* fat graft; *PG* plasma gel; *HA-1* hyaluronic acid filler 1 (Juvederm®); *HA-2* hyaluronic acid filler 2 (Teosyal®)

### Histopathological Findings of “Neovascularization”

**Fig. 9:**
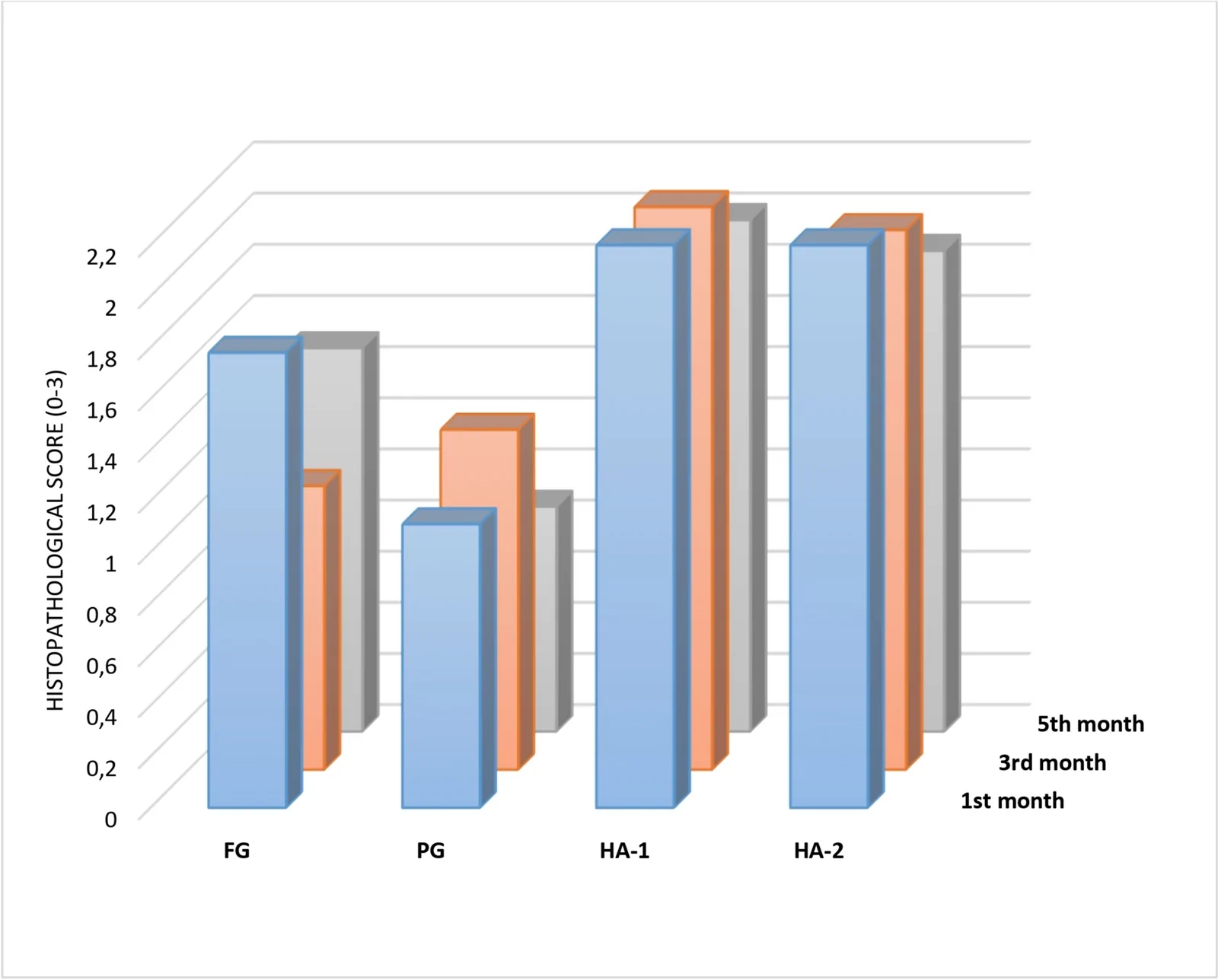
Comparison of histopathological “vascularization scores” across study groups at different time points

At 1 month, significant intergroup differences were observed (*p* < 0.001) (Fig. 9; Table 3). PG demonstrated significantly lower neovascularization compared with FG, HA-1, and HA-2. No significant difference was observed between HA-1 and HA-2.

**Table 3:** Histopathological evaluation of neovascularization at different time points

Time point	FG	PG	HA-1	HA-2	*p*-value
1 Month	1.78 ± 0.67 (1–3)	1.11 ± 0.33 (1–2)	2.44 ± 0.73 (1–3)	2.56 ± 0.53 (1–3)	< 0.001
3 Months	1.11 ± 0.33 (1–2)	1.33 ± 0.50 (1–2)	2.22 ± 0.44 (2–3)	2.11 ± 0.33 (2–3)	< 0.001
5 Months	1.50 ± 0.53 (1–2)	0.88 ± 0.35 (0–1)	2.00 ± 0.00 (2–2)	1.88 ± 0.35 (1–2)	< 0.001

^*^Values are expressed as mean ± standard deviation (minimum–maximum). Statistical comparisons were performed using appropriate non-parametric tests as indicated. A *p*-value < 0.05 was considered statistically significant. *FG* fat graft; *PG* plasma gel; *HA-1* hyaluronic acid filler 1 (Juvederm®); *HA-2* hyaluronic acid filler 2 (Teosyal®)

At 3 months, intergroup differences remained significant (*p* < 0.001). PG continued to demonstrate lower neovascularization compared with both HA fillers (*p* < 0.05), while no significant difference was observed between PG and FG.

At 5 months, significant differences persisted (*p* < 0.001). PG maintained lower neovascularization compared with HA-1 and HA-2, while showing comparable levels to FG.

### Histopathological Findings of “Inflammatory Response”

**Fig. 10:**
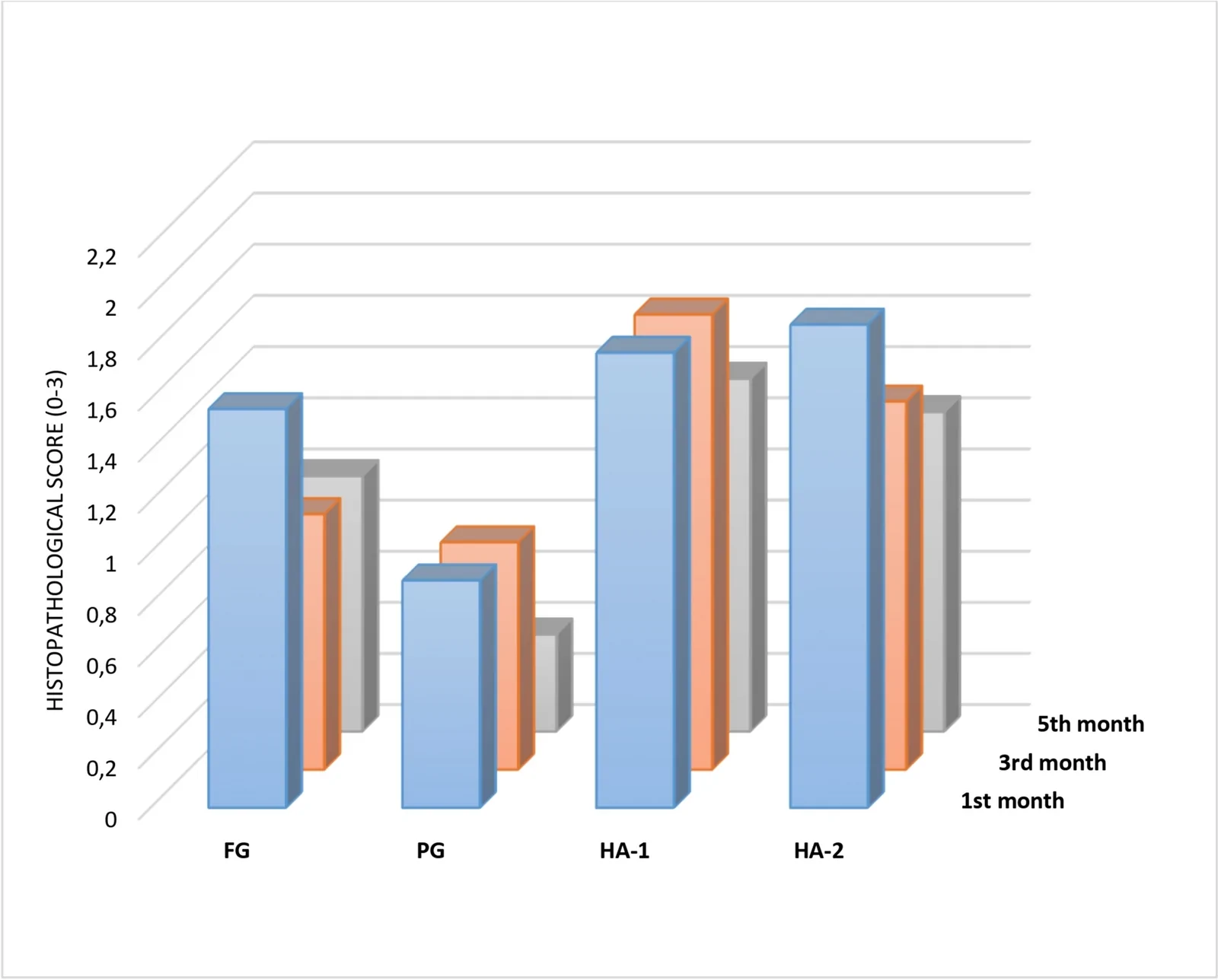
Comparison of histopathological “inflammation scores” across study groups at different time points

At 1 month, inflammatory response differed significantly among groups (*p* < 0.001) (Fig. 10; Table 4). PG demonstrated significantly lower inflammatory scores compared with FG, HA-1, and HA-2.

**Table 4:** Histopathological evaluation of inflammatory response at different time points

Time point	FG	PG	HA-1	HA-2	*p*-value
1 Month	1.56 ± 0.53 (1–2)	0.89 ± 0.33 (0–1)	1.78 ± 0.44 (1–2)	1.89 ± 0.78 (1–3)	< 0.001
3 Months	1.00 ± 0.00 (1–1)	0.89 ± 0.33 (0–1)	1.78 ± 0.67 (1–3)	1.44 ± 0.73 (1–3)	< 0.001
5 Months	1.00 ± 0.00 (1–1)	0.38 ± 0.52 (0–1)	1.38 ± 0.52 (1–2)	1.25 ± 0.46 (1–2)	< 0.001

^*^Values are expressed as mean ± standard deviation (minimum–maximum). Statistical comparisons were performed using appropriate non-parametric tests as indicated. A *p*-value < 0.05 was considered statistically significant. *FG* fat graft; *PG* plasma gel; *HA-1* hyaluronic acid filler 1 (Juvederm®); *HA-2* hyaluronic acid filler 2 (Teosyal®)

At 3 months, intergroup differences remained significant (*p* < 0.001). PG continued to exhibit lower inflammatory response compared with both HA fillers (*p* < 0.05), while no significant difference was observed between PG and FG.

At 5 months, significant differences persisted (*p* < 0.001). PG demonstrated the lowest inflammatory response among all groups. Compared with FG, a significant reduction in inflammation was observed (*p* < 0.05), and PG continued to show significantly lower inflammatory scores compared with HA-1 and HA-2.

## Discussion

The present experimental study evaluated the volumetric behavior and tissue response of PG in comparison with FG and hyaluronic acid fillers in a controlled animal model. PG demonstrated lower volume at 1–3 months compared with FG and hyaluronic acid fillers. However, by 5 months, no statistically significant differences were observed among the groups. These findings suggest that although PG provides less early volume augmentation, its long-term volumetric persistence becomes comparable to established fillers. The lower early volume may be related to the absence of cross-linking and structural stabilization mechanisms present in hyaluronic acid fillers [5, 11].

In this model, PG demonstrated a more stable longitudinal volumetric profile compared with FG, while achieving comparable outcomes to hyaluronic acid fillers at later time points. Importantly, this was accompanied by a more favorable tissue response, with consistently lower inflammatory and neovascularization scores. These findings suggest that PG induces a milder local tissue reaction than FG and hyaluronic acid–based fillers, which may contribute to its volumetric stability over time.

Previous studies have investigated plasma-derived materials, including PG, as autologous bio-fillers, while others have compared hyaluronic acid fillers and fat-based injectables in animal models [9, 12]. However, these studies have generally focused on single comparisons or short-term outcomes [12–14]. The within-subject design of the present study is a key strength, enabling direct comparison of all materials under identical biological conditions while minimizing inter-animal variability. In addition, the use of CT-based volumetric analysis and histopathological evaluation provides a comprehensive assessment of both structural and biological behavior.

From a translational perspective, the rat subcutaneous injection model is widely used in preclinical filler research to evaluate volumetric behavior, tissue integration, and inflammatory response [12, 15]. Although anatomical and biomechanical differences between rat dorsal tissue and human facial soft tissues exist, this model provides a controlled and reproducible platform for comparative evaluation under identical conditions [16]. Within this context, the findings suggest that PG may be a suitable option in applications where biocompatibility and minimal tissue reaction are prioritized. However, its lower early volumetric performance should be considered in clinical planning.

**Fig. 11:**
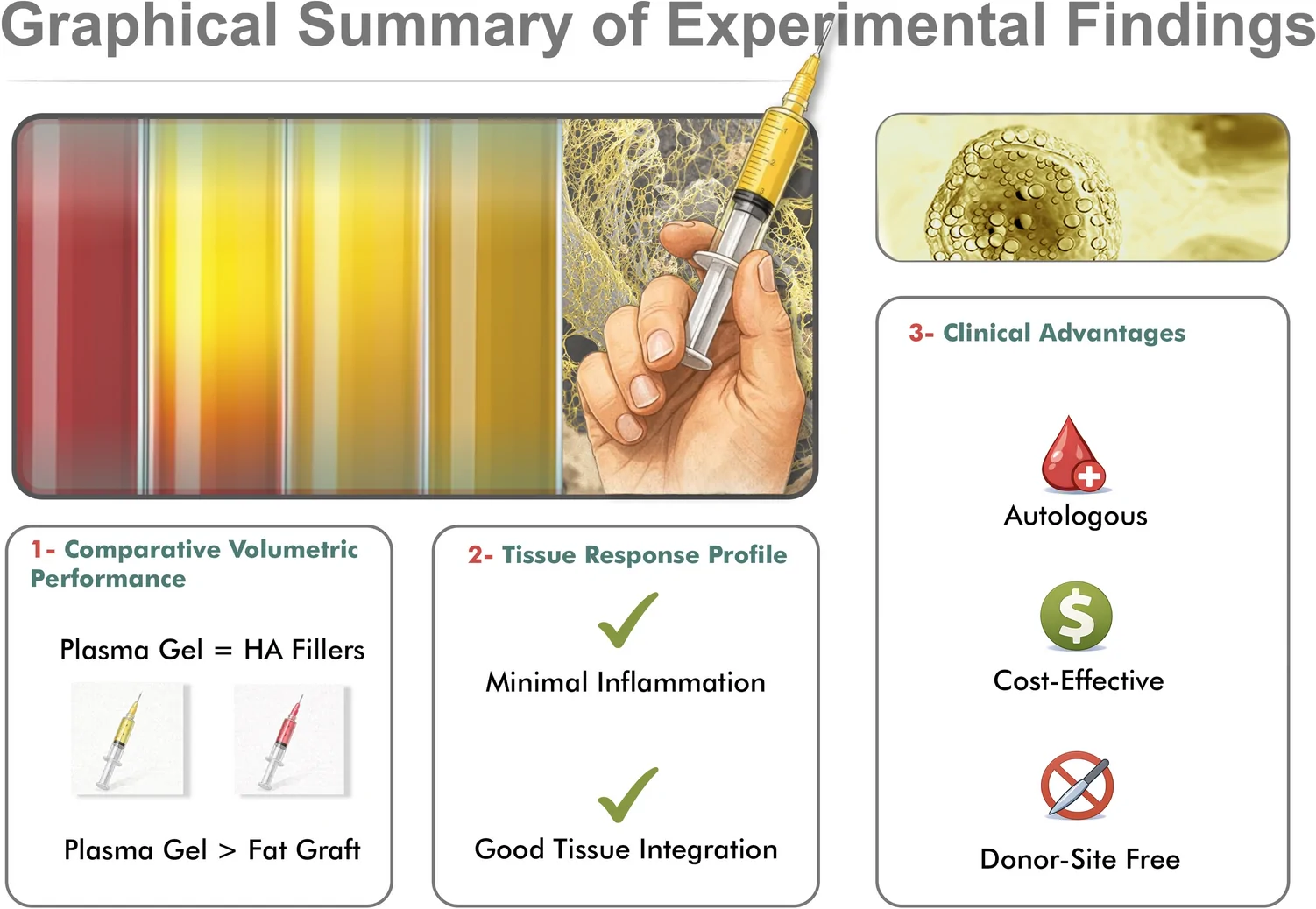
Graphical summary of the main experimental findings. Graphical summary illustrating the principal biological and clinical features of autologous plasma gel (PG), including favorable volumetric behavior in comparison with control fillers, minimal inflammatory response, good tissue integration, autologous origin, cost-effectiveness, and absence of donor-site morbidity

Given its autologous origin and consistently low inflammatory response, PG demonstrates favorable biocompatibility, which may be advantageous in settings where minimizing tissue reaction is critical (Fig. 11). Nevertheless, these findings are derived from a preclinical model and should be interpreted cautiously until validated by clinical studies.

Commercial hyaluronic acid fillers served as a reliable benchmark in this study and demonstrated the predictable volumetric behavior expected from established clinical standards [2]. The comparable volumetric performance of PG supports its potential role as a temporary volumizing option in selected aesthetic settings, particularly when an autologous approach is preferred.

Although molecular or biochemical characterization of PG was not performed, the consistently lower inflammatory and neovascularization scores suggest a more stable tissue response. This reduced reaction may partially explain the favorable volumetric maintenance observed over time and supports further clinical investigation of PG.

This study has several limitations. Molecular and biochemical characterization was not performed, and the findings are based on an animal model with inherent differences from human soft tissues. Therefore, clinical studies are needed to validate these results. Additional macroscopic images and material characteristics are provided in the Supplemental Content.

## Conclusion

PG appears to be a safe and reproducible autologous filler with a favorable biological profile. Although early volumetric effect is lower, long-term volume retention becomes comparable to hyaluronic acid fillers and FG, with consistently reduced inflammatory and neovascularization responses.

These findings suggest that PG may be a promising temporary filler option in clinical settings where biocompatibility and minimal tissue reaction are prioritized. Further clinical studies are required to confirm its safety and efficacy in humans.

## Supplementary Information

Below is the link to the electronic supplementary material.

Macroscopic appearance of the dorsal region following injection, demonstrating distinct subcutaneous elevations corresponding to each injected material.(JPG 3393 KB)

Autologous plasma gel (PG) in a syringe before injection, demonstrating a homogeneous and cohesive structure following thermal processing. (JPG 1446 KB)
